# Safety and immunogenicity of a recombinant double-mutant heat-labile toxin derived from enterotoxigenic *Escherichia coli* in healthy Bangladeshi adults delivered by three different routes

**DOI:** 10.3389/fbrio.2025.1567791

**Published:** 2025-07-15

**Authors:** Taufiqur Rahman Bhuiyan, Farhana Khanam, Salima Raiyan Basher, Pinki Dash, Mohiul Islam Chowdhury, Shahinur Haque, Nabila Binte Harun, Aklima Akter, Polash Chandra Karmakar, Al Hakim, Shaheena Amin, Mohammad Kamruzzaman, Nasrin Parvin, Tasnuva Ahmed, Jessica Butts, Marcela F. Pasetti, Rezwanul Wahid, Marcelo B. Sztein, Nicole Maier, Jessica A. White, Kay M. Tomashek, A. Louis Bourgeois, Shahida Baqar, Karen L. Kotloff, Firdausi Qadri, Wilbur H. Chen

**Affiliations:** 1 Infectious Diseases Division, International Centre for Diarrhoeal Disease Research, Bangladesh (icddr,b), Dhaka, Bangladesh; 2 The Emmes Company, LLC, Rockville, MD, United States; 3 Center for Vaccine Development and Global Health, University of Maryland School of Medicine, Baltimore, MD, United States; 4 PATH, Washington, DC, United States; 5 Division of Microbiology and Infectious Diseases, National Institute of Allergy and Infectious Diseases, National Institutes of Health, Bethesda, MD, United States

**Keywords:** enterotoxigenic *Escherichia coli*, diarrhea, vaccine, adjuvant, double-mutant heat-labile toxin

## Abstract

**Introduction:**

Enterotoxigenic *Escherichia coli* (ETEC) is a common cause of acute watery diarrhea in areas lacking access to clean water, sanitation, and hygiene. This Phase 1 trial measured the safety and immunogenicity of double-mutant heat-labile enterotoxin (dmLT) of ETEC in healthy adults in Bangladesh, where ETEC is endemic.

**Methods:**

Five cohorts of 15 participants each were enrolled and randomized 4:1 to receive vaccine dmLT or placebo (12 vaccine and 3 placebo recipients per cohort). The 3 oral or sublingual doses of 5 μg or 25 μg dmLT were administered 2 weeks apart; the 2 intradermal doses of 0.3 μg dmLT were administered 3 weeks apart. Safety was assessed by collecting solicited and unsolicited adverse events. The immune responses measured included dmLT-specific serum IgA and IgG, serum toxin neutralizing antibody, dmLT-specific IgA and IgG antibody secreting cells (ASC), and IgA and IgG antibodies in lymphocyte supernatant (ALS).

**Results:**

All doses of dmLT delivered by different routes were well tolerated; adverse events were few, mild, and transient. Serum, ALS, and ASC IgA and IgG responses, as well as LT neutralizing antibody responses, were greatest among recipients of 25 μg oral and 0.3 μg intradermal doses. In contrast, sublingual dosing induced modest responses; there was virtually no serum antibody response to 5 μg sublingual dose and only sporadic ALS and ASC responses with 5 μg and 25 μg doses.

**Discussion:**

In conclusion, dmLT was well tolerated, and immune responses were dependent on dmLT dose and route of administration. The encouraging tolerability and immunogenicity results further highlight dmLT’s potential not only as a vaccine but also as an adjuvant as reported by others or as a candidate vaccine antigen.

**Clinical Trial Registration:**

www.clinicaltrials.gov, identifier NCT03548064.

## 1 Introduction

Enterotoxigenic *Escherichia coli* (ETEC) is a major causative agent of acute diarrhea globally, estimated to be responsible for approximately 220 million annual diarrheal episodes and 42,000 deaths per year among children under 5 years of age (Lanata et al., 2013; Khalil et al., 2018). However, there is no licensed vaccine to prevent illness caused by ETEC (Riddle et al., 2018). Antigens that have been targeted for vaccine development include various colonization factors, and either a poorly immunogenic, short peptide heat-stable toxin (ST) that appears to confer considerable virulence and/or the highly, but transiently immunogenic heat-labile toxin (LT).

LT is an oligomeric protein consisting of a single enzymatically active A-subunit and five identical receptor binding B-subunits that attach to the epithelial cell surface. LT upregulates cAMP (cyclic adenosine monophosphate) and causes active secretion of water and electrolytes into the lumen of the small intestine, thereby resulting in secretory diarrhea which can be voluminous (Sears and Kaper, 1996; El-Kamary et al., 2013). Recent data also indicates that LT drives a number of enteropathic changes in the small intestinal epithelia that can potently contribute to both the acute and more long-term negative health consequences of ETEC infection in infants and children in low- and middle-income countries (LMICs) and appears to have an adjuvant effect when administered concomitantly with other mucosal antigens (Sheikh et al., 2020; Sheikh et al., 2022). In addition, the increases in cAMP linked to LT may also be associated with other cellular changes and receptor expression in the intestine that may impact pathogen-host interactions related to ETEC and other enteropathogens (Allen et al., 2006; Glenn et al., 2009; Verbrugghe et al., 2015; Sheikh et al., 2020).

The favorable properties of LT as a potential vaccine antigen and mucosal adjuvant have prompted efforts to eliminate its enterotoxicity (enzymatic effect) while maintaining its immunogenicity. A single mutation in the A subunit to create mLT or LT(R192G) protein resulted in marked attenuation but residual enterotoxicity was observed (Lycke et al., 1992; Dickinson and Clements, 1995; Kotloff et al., 2001; Lapa et al., 2008). A second-generation derivative, double-mutant LT [dmLT or LT(R192G/L211A)] was created with no enterotoxicity for further human study evaluation (Norton et al., 2011). For comparison, only 5 μg of native LT is necessary to induce diarrhea in humans (Levine et al., 1983) whereas in an earlier study up to 25 μg (the highest dose tested) of dmLT given orally was non-reactogenic in humans (Bernstein et al., 2019). The successful attenuation of LT made further clinical development possible (El-Kamary et al., 2013; Bernstein et al., 2019). These studies highlight the potential value of dmLT as an antigen for inclusion in candidate ETEC vaccines.

Furthermore, the use of dmLT as a mucosal adjuvant has been explored. The coadministration of dmLT with a live, oral, attenuated ETEC vaccine candidate, ACE527, induced a strongly protective immune response among U.S. adults in a controlled human infection model, CHIM (Harro et al., 2019). The addition of dmLT was also evaluated among Bangladeshi adults for an oral inactivated ETEC vaccine candidate, ETVAX; although there was not a statistically significant adjuvant effect of dmLT on the antibody responses to the ETEC antigens, the overall antigenic breadth of the plasma IgA response tended to favor the adjuvanted vaccine (Akhtar et al., 2019). In the first evaluation of dmLT in infants and children, Bangladeshi children and infants demonstrated trends for improved mucosal immune responses to ETVAX (Qadri et al., 2020). Infants demonstrated significantly improved mucosal responses to 3 of the 5 colonization factor antigens in the vaccine, as well as, to the O78 LPS expressed on 3 of the ETEC strains included in the vaccine (Svennerholm et al., 2022). In follow-up studies, dmLT also improved the expression of B cell memory markers in adults and T cell responses in Bangladeshi adults and infants (Akhtar et al., 2021; Mottram et al., 2021). Fractional doses of inactivated polio vaccine combined with dmLT and administered intradermally also demonstrated improved serum neutralizing antibody response (Crothers et al., 2022). In parallel studies of an early stage ETEC subunit vaccine, initially, mLT and more recently, dmLT were well tolerated when given by the ID (intradermal) and IM (intramuscular) routes, induced strong anti-LT immunes responses, and also significantly enhanced the serum and mucosal responses to the co-administered ETEC colonization factors antigens (Lee et al., 2021; Gutiérrez et al., 2023). In the ID study, immunization with the CfaE ETEC adhesin and mLT was also shown to reduce the incidence and severity of ETEC diarrhea following challenge with ETEC strain H10407 (Gutiérrez et al., 2024). These studies further document the safety and potential of using dmLT as both a key antigen and adjuvant in ETEC and other enteric vaccines.

Nonetheless, strategic questions remain regarding the immunogenicity of dmLT: firstly, the required dose of dmLT as an isolated immunogen for an immunologically primed population (i.e., a population endemic for ETEC infections) needs to be determined; secondly, the impact of the route of administration (oral, sublingual, or intradermal) in the frequency and/or functionality of responses needs to be explored. In earlier preclinical studies, delivery and formulation parameters have been shown to help shape the adaptive immune response to both dmLT and co-administered ETEC antigens (Maciel et al., 2019). This study aims to characterize the safety and immunogenicity of dmLT when administered orally, sublingually (SL), and intradermally (ID) in healthy adults of an ETEC endemic country. These data will inform the antigen-specific responses among previously primed individuals and better guide how dmLT may be used as both a vaccine antigen and adjuvant in future vaccine candidates.

## 2 Materials and methods

### 2.1 Vaccine

The dmLT was produced according to cGMP (current Good Manufacturing Practices) specifications by IDT Biologika and was formulated as a lyophilized product, containing ~500 mg of vaccine protein in 2 mL sterile vials, to be stored at −20°C. The dmLT (Lot 001–08-15) has been on a stability plan since the initial lot release in 2016.

### 2.2 Study design

Healthy adults 18 to 45 years of age from the Mirpur area of Dhaka, Bangladesh were recruited to participate in this double-blinded, placebo-controlled randomized, dose-ascending, Phase 1 trial. Impartial literate witnesses to the informed consent process were provided when participants were illiterate. Consent forms were translated into Bengali, and the study was approved by both the local Ethics Board of the International Centre for Diarrhoeal Disease Research, Bangladesh (icddr,b, Dhaka, Bangladesh) and the University of Maryland, Baltimore Institutional Review Board.

Eligible participants were non-pregnant, healthy volunteers who provided written informed consent, had no recent moderate or severe diarrheal illness (within 6 weeks of enrollment), and satisfied the protocol-defined inclusion and exclusion criteria (registered in https://clinicaltrials.gov/ct2/show/NCT03548064). They were enrolled into 5 sequential cohorts, each consisting of 15 participants who were randomized 4:1 to dmLT versus placebo with the dmLT dosing as follows: 5 μg orally, 25 μg orally, 5 μg SL, 25 μg SL, or 0.3 μg ID. Participants received three spaced doses on Days 1, 15, and 29 for the oral and SL routes and on Days 1 and 22 for the ID route.

Participants were observed fasting for at least 90 minutes before and after oral or 30 minutes before and after SL vaccination; ID vaccination did not require fasting. Oral vaccination consisted of the ingestion of 120 mL of bicarbonate solution (2 g NaHCO_3_/150 mL H_2_O), followed 1–5 minutes later by the ingestion of 30 mL of bicarbonate buffer solution containing the specified dose of dmLT. SL vaccination was preceded by mouth rinsing and gargling for 10 minutes with bottled water, then the placement of a sterile gauze under the tongue for 1 minute. The designated SL dose of dmLT, diluted with sterile saline, was given in a 100 μL volume using a 1 mL syringe, after which participants tilted their heads forward (chin to chest) for 1 minute avoiding swallowing. Prior to ID vaccination, the injection site was examined for scars, tattoos, or any abrasion that might make the evaluation of local reactogenicity difficult. The designated dose of dmLT was delivered in a 100 μL volume tuberculin syringe with a 25-gauge needle. Dose verification was performed for each dosing cohort. Placebo consisted of bicarbonate buffer for blinded oral doses and consisted of sterile normal saline for blinded SL and ID doses. The study was interrupted by the COVID-19 pandemic and prevented the evaluation of dose escalation of oral, SL, and ID dmLT, as originally planned.

Participants were instructed to complete a memory aid to report solicited systemic and local reactions for 7 days after each dose of vaccine or placebo. Follow-up clinic visits were completed on Days 8, 22, and 36 (oral and SL) or Days 8, and 29 (ID) for the collection and review of the memory aids. Additional follow-up clinic visits were also completed 4 weeks, 12 weeks, and 6 months (except ID cohort) following their third dose of blinded study product.

### 2.3 Laboratory assays

#### 2.3.1 Serum antibodies

Serum dmLT-specific IgA and IgG were measured by ELISA (Enzyme-linked immunosorbent assay) as previously described (Tacket et al., 2004; El-Kamary et al., 2013) The titers (EU/mL) were calculated through linear regression curves as the inverse of the highest serum dilution that produces an optical density (OD)_450 nm_ of 0.2 above the mean of the blanks. Seroconversion was defined as an antibody titer increase of ≥ 4-fold over baseline. For Serum ELISA and LT neutralization assays, samples were collected on Days 1, 8, 15, 22, 29, 36, 57, and 114 for oral and SL Cohorts and Days 1, 8, 22, and 29 for ID Cohort. Due to the COVID-19 pandemic, serum samples were not collected on day 114 from participants receiving 25 μg of dmLT by SL route.

#### 2.3.2 LT neutralization

LT-toxin neutralizing antibodies were measured, as previously described (Frech et al., 2007; Bernstein et al., 2019). Briefly, serially diluted samples were incubated with 5 ng/mL of LT (Berna Biotech, Berne, Switzerland) for 30 minutes at 37°C. Y-1 Adrenal Cells (ATCC, Manassas, VA) were added at 2.5×10^4^ cells/well, and plates were incubated for 15–18 hours at 37°C. Endpoint titers were reported as the reciprocal of the highest serum dilution that resulted in ≥ 50% reduction of cell rounding. A positive response was defined as a ≥ 4 -fold increase in titer over baseline.

#### 2.3.3 Antibody secreting cells

Circulating IgG and IgA dmLT-specific ASCs were measured by ELISpot using freshly isolated peripheral blood mononuclear cells (PBMC) using published methods (Tacket et al., 2004; El-Kamary et al., 2013; Dash et al., 2024). The frequency of ASC was expressed as the number of IgA or IgG spot forming cells (SFC) per 10^6^ PBMC. A positive ASC response was defined as ≥8 SFC per 10^6^ cells. As a part of the quality control of our experiment, Total Immunoglobulin (TIg) was used in the assay. For the ASC assay, samples were collected on Days 1, 8, 15, 22, 29, and 36 for oral and SL Cohorts and on Days 1, 8, 22, and 29 for the ID Cohort.

#### 2.3.4 Antibodies in lymphocyte supernatants

The ALS assay quantifies the dmLT-specific IgA and IgG antibody secreted *in vitro* from tissue cultures of PBMCs (1×10^7^ cells/mL in complete RPMI) on similar time points as ASC assay and incubated with dmLT for 72 hours as previously described (El-Kamary et al., 2013). Briefly, culture supernatants were collected and stored at −20°C until tested by ELISA for the presence of dmLT-specific antibodies. A positive ALS response was defined as a≥ 2-fold increase over baseline.

### 2.4 Statistical analysis and sample size

Although no formal sample size calculation was performed, the number of participants was selected to be appropriate for a Phase 1 study. AEs (adverse events) and reactogenicity are summarized using the number and percentage of participants who experienced each event overall. The immunogenicity analyses are reported from the modified intent to treat (mITT) population, defined as participants who received at least one dose of study product and contributed both pre- and at least one post-vaccination sample for testing for which valid results were reported. Antibody titers were compared within and between groups by Wilcoxon signed rank test and Mann-Whitney test respectively. All reported p-values are two-sided using the 0.05 level of significance. No corrections for multiple comparisons were applied. All data analyses and statistical computations were conducted with SAS software (version 9.4) or GraphPad Prism (version 8).

#### 2.4.1 Linear mixed model analysis

The possible correlations of the serum LT neutralizing antibody responses with other immune responses in participants receiving dmLT were explored using linear mixed models. All immune responses were log_2_-transformed, and the ASC values of 0 were imputed as 1 prior to calculating the log to avoid mathematical errors. Models were fit separately for each route of administration. For the oral and SL routes, an interaction model containing fixed effects for serum LT neutralizing antibody, dose group, and their interaction was fit first. If the interaction term was not significant, a main effects model was fit without the interaction term. For the intradermal route, there was only one dose group, so a single main effects model was fit with serum LT neutralizing antibody as the only fixed effect. All available time points were included in the models. All models were estimated using restricted maximum likelihood. The empirical variance-covariance estimator was used to adjust the standard errors for hypothesis tests. The p-values are from Type III tests of fixed effects using an F statistic, using the containment method to compute the denominator degrees of freedom. Models were fit using proc mixed. Two R^2^ values were calculated, marginal R^2^ (R_m_^2^) was the proportion of the total variation that is explained by the fixed effects alone and conditional R^2^ (R_c_^2^) was the proportion of the total variation explained by the fixed and random effects (Nakagawa and Schielzeth, 2013).

## 3 Results

### 3.1 Study design

**FIGURE 1:**
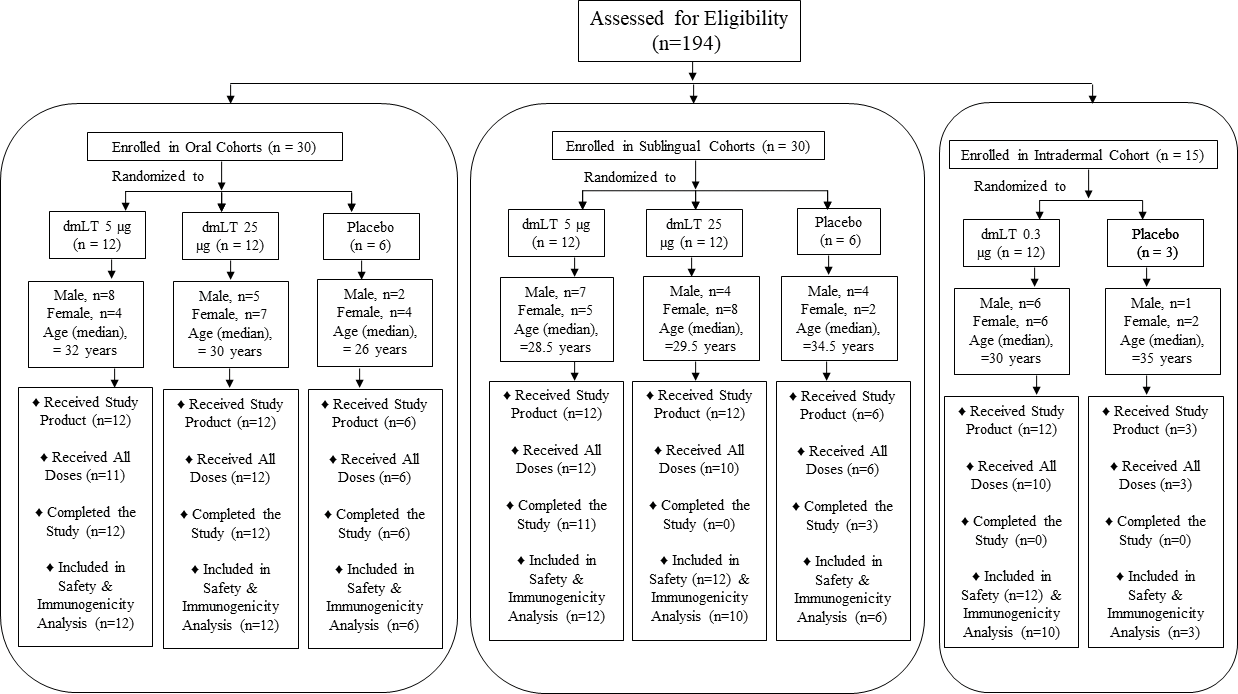
194 healthy participants were screened, of which 75 were enrolled in 5 cohorts receiving dmLT in three different routes: 30 in oral route, 30 in sublingual route and 15 in intradermal route. In each group of oral and sublingual route, 12 participants received 5 μg and another 12 received 25 μg dmLT and 6 were placebo. In intradermal group, 12 received 0.3 μg dmLT and 3 were placebo. Number of male and female participants are presented.

A total of 194 individuals were screened, 109 were determined to be eligible for enrollment, and 75 were enrolled from 10 March 2019 through 11 February 2020 (Figure 1). Out of 75 participants enrolled, 59% (n=44) completed the study and 41% (n=31) did not complete all follow-up visits: of these 31, 1 participant withdrew voluntarily (SL 5 μg dmLT group), and 30 participants could not complete follow up due to the closures during the COVID-19 pandemic (12 in the SL 25 μg dmLT group and 3 in the SL placebo group, 12 in the ID 0.3 μg dmLT group and 3 in the ID placebo group). Of the 75 enrolled participants, 18 (24%) discontinued study vaccination: 1 in the oral 5 μg dmLT group due to pregnancy, 2 in the SL 25 μg dmLT group who were lost to follow-up, and 12 in the ID 0.3 μg dmLT group and 3 in the ID placebo group none of whom could receive their scheduled third dose due to the closures during the COVID-19 pandemic.

### 3.2 Safety

Out of 75 participants enrolled in the study, 8 (11%) experienced at least one systemic solicited AE, and 6 (8%) experienced at least one local solicited AE (Table 1). All the solicited AEs were graded as mild. Six (Sheikh et al., 2022) participants (8%) experienced at least one unsolicited AE, and 2 participants (3%) experienced at least one unsolicited AE related to the study product. Six (Sheikh et al., 2022) participants (8%) experienced at least one clinical safety laboratory AE. All clinical safety laboratory AEs were graded as mild. No deaths, serious adverse events, or AEs leading to early termination were reported.

### 3.3 Immunogenicity

Vaccine induced immune responses were examined by measurement of dmLT-specific IgA and IgG and LT neutralizing antibodies in serum, frequency of dmLT-specific IgG and IgA ASCs, and IgG and IgA ALS.

#### 3.3.1 Serum antibody responses

**FIGURE 2:**
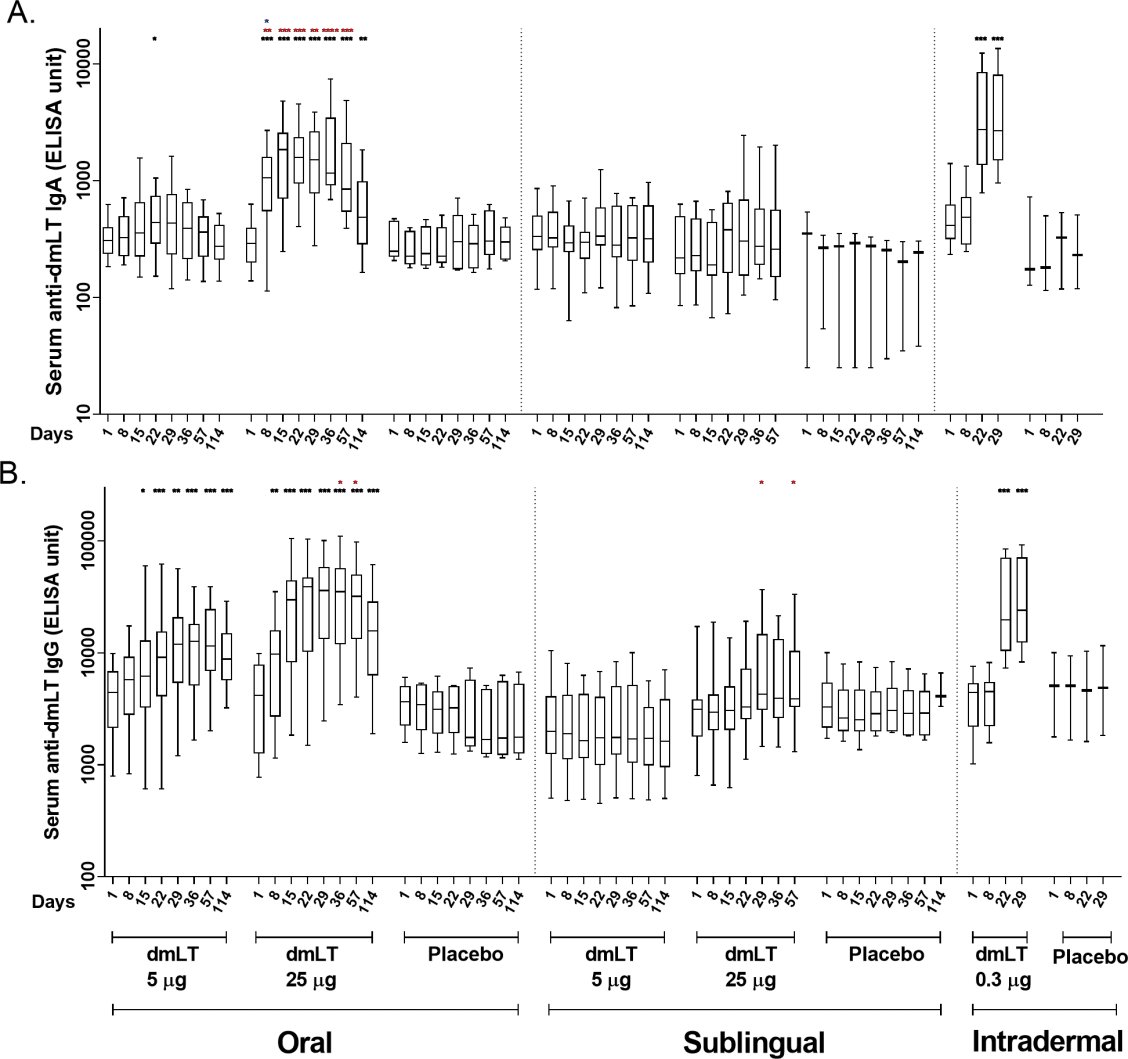
Serum antibody responses to dmLT antigen in vaccinees and placebo participants in three different routes. **(A)** Immunoglobulin A (IgA) antibody responses and **(B)** IgG antibody responses to dmLT in Oral, Sublingual and Intradermal route following vaccination in vaccinees and placebo. The Wilcoxon signed-rank test was used for analysis of the data within group. Black asterisks indicate a statistically significant difference in titer from baseline level. Mann Whitney test was used for analysis of the data between groups. Red asterisks indicate a statistically significant difference between titer of the same day of two groups, receiving the vaccine through the same route of administration. Blue asterisks indicate a statistically significant difference between titer of a particular day of oral 25 μg and intradermal 0.3 μg vaccinees. (***P < 0.001, **P <0.01, *P <0.05). Median and interquartile ranges are also presented.

Anti-dmLT serum IgA and IgG responses are shown in Figure 2. A significant increase in serum IgA response was observed at day 22 when compared to baseline, in participants receiving 5 μg dmLT orally (Figure 2A). A significant increase in IgG response was observed at days 15, 22, 29, 36, 57, and 114 in comparison to baseline in the same group (Figure 2B). Those who received 25 μg dmLT orally showed significantly increased IgA and IgG responses at all post-baseline time points compared to baseline (4–7 fold rise for IgA and 2–9 fold rise for IgG). Significantly 6–8 fold increase in IgA and IgG responses were observed at days 22 and 29 compared to baseline in the group who received 0.3 μg dmLT intradermally.

We further analyzed the differences between the two groups receiving 5 μg and 25 μg of dmLT orally at the same days. An elevated IgA titer was observed at days 8, 15, 22, 29, 36, and 57, and again an elevated IgG titer was observed at days 36, and 57 in the 25 μg oral dmLT group in comparison to the 5 μg oral dmLT group. An increased IgA response was observed at day 8 in oral 25 μg group in comparison to that of the intradermal 0.3 μg group.

The anti-dmLT serum IgA and IgG geometric mean titer (GMT) and 95% confidence intervals are presented in Supplementary Table 1. The serum IgG and IgA responses elicited by the oral dmLT groups followed a dose-response pattern with a 50% responder rate compared to a 100% responder rate by serum IgG and a 17% responder rate compared to 92% responder rate by serum IgA in the 5 μg and 25 μg oral dmLT groups respectively. There was a less pronounced dose-response relationship in the SL dmLT groups with a 0% response rate compared to a 20% response rate for both IgG and IgA in the 5 μg and 25 μg oral dmLT groups respectively. The 0.3 μg ID dmLT group showed a 75% and 83% responder rate by serum IgG and IgA respectively. Antigen-specific serum IgA and IgG responses peaked after the third dose of oral 25 μg dmLT and after the second dose of ID 0.3 μg dmLT which were the last respective doses of the vaccine. The maximum GMTs for IgG across all groups occurred at Day 29 in the 25 μg oral dmLT group (GMT = 27326, 95% CI = 13827, 53965) and in the 0.3 μg ID dmLT group (GMT = 27172, 95% CI = 15821, 46669), or 14 and 7 days respectively after the second oral and ID doses were administrated. The maximum GMT for IgA across all groups occurred at Day 29 or seven days after the second 0.3 μg ID dose was administered (GMT = 3344, 95% CI = 1838, 6084).

#### 3.3.2 Serum LT neutralizing antibody responses

Serum LT neutralizing antibody responses elicited by routes and doses are shown in Table 2. All recipients of the 25 μg oral dose and 92% of those who received the 5 μg oral dose seroconverted. A high rate of seroconversion (83%) was also seen among participants immunized intradermally with 0.3 μg of dmLT. In contrast, very few participants receiving SL dmLT seroconverted with seroconversion rates of 20% and 0% in the 25 μg and 5 μg dose groups respectively. The 25 μg oral dmLT group exhibited the highest GMTs, which remained elevated through day 114, followed by the 0.3 μg ID dmLT group who had elevated GMTs at days 22 and 29 although further follow-up was interrupted by COVID-19. Responses were low in participants receiving SL dmLT when compared to the oral and ID groups. No response was seen in the placebo recipients.

#### 3.3.3 Circulating antibody-secreting cell responses

**FIGURE 3:**
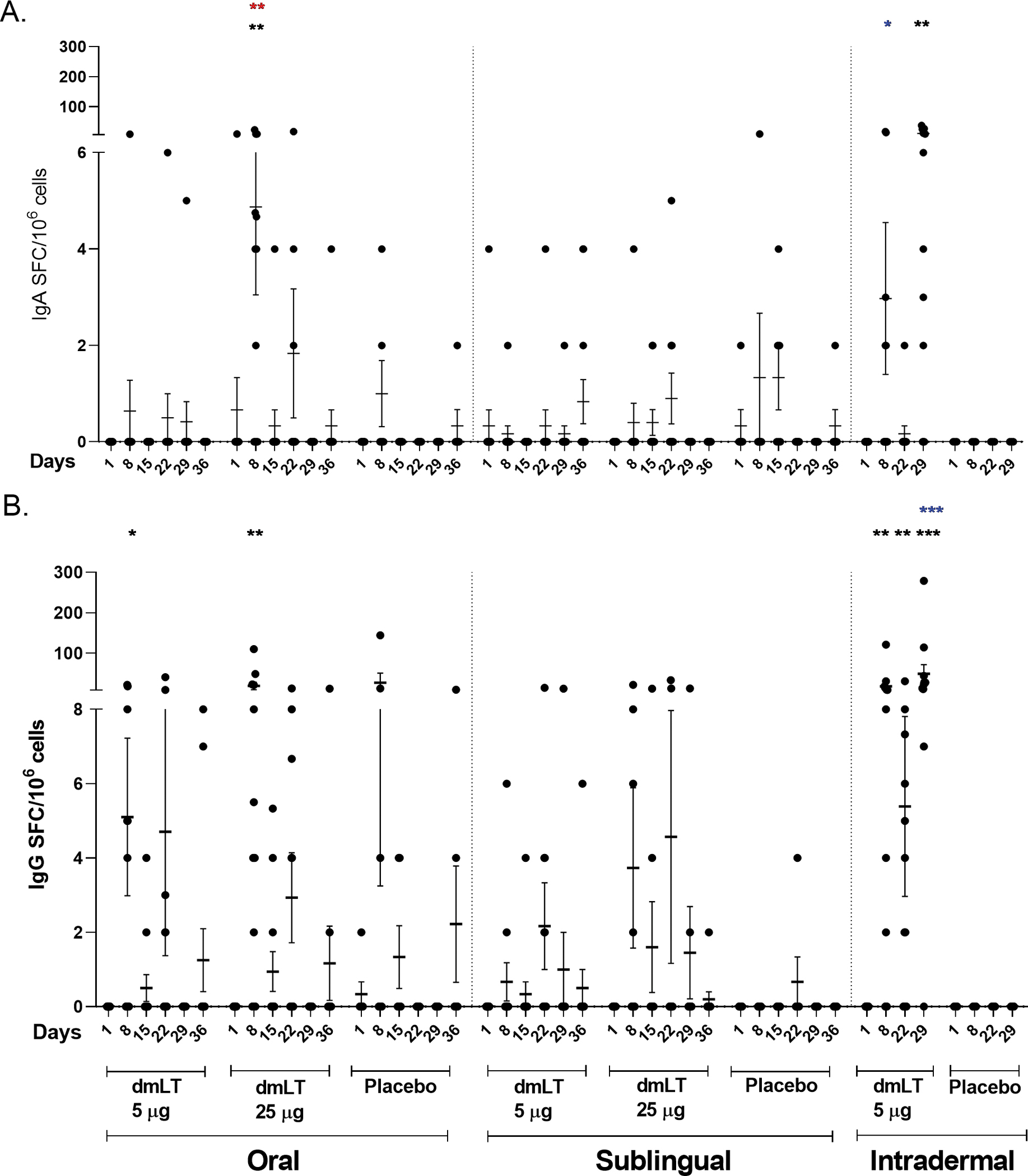
dmLT specific antibody secreting cell (ASC) responses in vaccinees and placebo participants in three different routes. Mean ± standard errors of the mean (SEM) of the circulating **(A)** Immunoglobulin A (IgA) and **(B)** IgG antibody secreting cells to dmLT in Oral, Sublingual and Intradermal route are represented. Each dot represents dmLT specific spot forming cells per million. The Wilcoxon signed-rank test was used for analysis of the data within group. Black asterisks indicate a statistically significant difference from baseline level, Day 1. Mann Whitney test was used for analysis of the data between the groups. Red asterisks indicate a statistically significant difference between ASC responses of the same day point of two groups, receiving the vaccine through the same route of administration. Blue asterisks indicate a statistically significant difference between ASC responses of a particular day point of oral 25 μg and intradermal 0.3 μg vaccinees. (***P < 0.001, **P <0.01, *P <0.05).

The presence of circulating dmLT-specific IgA and IgG ASCs were measured for each dose group (Figure 3). For the oral route, a significant increase in IgA ASC response was observed at day 8 compared to baseline in vaccinees receiving 25 μg dmLT, and the IgG ASC response was significantly increased at day 8 in both the 5 μg and 25 μg dmLT groups compared to their respective baselines. For the ID route, the dmLT-specific IgA ASC response was significantly higher on day 29 compared to baseline, and IgG ASC response was significantly higher on days 8, 22, and 29 compared to baseline. There was a significant elevation of IgA ASC response at day 8 in the participants receiving 25 μg oral dmLT compared to those receiving 5 μg oral dmLT. There was a significant difference between the 0.3 μg ID dmLT and 25 μg oral dmLT groups at Day 8 for IgA and Day 29 for IgG where responses were higher in 25 μg oral dmLT group.

The dmLT-specific ASC response rates were 50% and 67% for IgG and 0% and 25% for IgA in the 5 μg and 25 μg oral dmLT groups respectively. For the SL route, the response rates were 17% and 30% for IgG and 8% and 0% for IgA in the 5 μg and 25 μg dose groups respectively. There were 83% and 52% responders in the 0.3 μg ID dmLT group for IgG and IgA respectively (Supplementary Table 2). The highest percentage of ASC responders was observed in the ID 0.3 μg group followed by the oral 25 μg group.

#### 3.3.4 ALS assay

**FIGURE 4:**
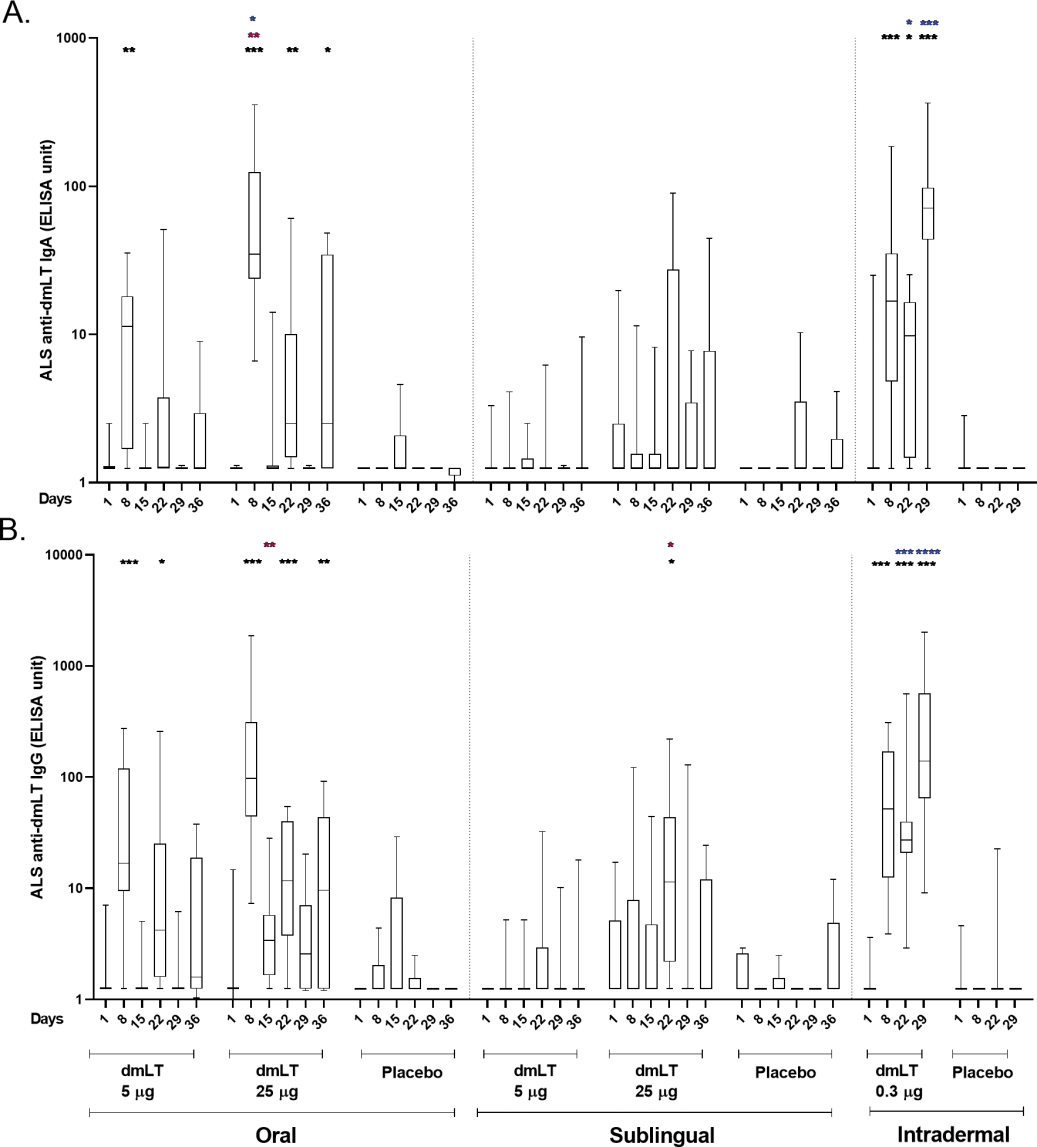
dmLT specific ALS responses in vaccinees and placebo participants in three different routes. **(A)** Immunoglobulin A (IgA) antibody responses and **(B)** IgG antibody responses to dmLT in Oral, Sublingual and Intradermal route following vaccination in vaccinees and placebo. The Wilcoxon signed-rank test was used for analysis of the data within group. Black asterisks indicate a statistically significant difference in titer from baseline level. Mann Whitney test was used for analysis of the data between the groups. Red asterisks indicate a statistically significant difference between titers of the same time points of two groups, receiving the vaccine through the same route of administration. Blue asterisks indicate a statistically significant difference between titer of a particular day point of oral 25 μg and intradermal 0.3 μg vaccinees. (***P < 0.001, **P <0.01, *P <0.05). Median and interquartile ranges are also presented.

The dmLT-specific IgA and IgG ALS responses are presented in Figure 4. In the oral 5 μg dmLT group, there were elevated responses compared to baseline for IgA at day 8 and for IgG at days 8 and 22. In oral 25 μg dmLT group, both IgA and IgG responses were significantly increased compared to baseline at days 8, 22, and 36. For the sublingual 25 μg dmLT group, IgG response was significantly higher at day 22 in comparison to baseline.

A significantly higher response was noted on day 8 for IgA and on day 15 for IgG in the 25 μg oral dmLT group compared to the 5 μg oral dmLT group. Both IgA and IgG responses were significantly higher on days 22 and 29 in the 0.3 μg ID dmLT group compared to the 25 μg oral dmLT group. However, on day 8, responses were significantly higher in the 25 μg oral dmLT group when compared to the 0.3 μg ID group.

The dmLT-specific ALS response rates were high in both the 5 μg and 25 μg oral dmLT groups with response rates of 92% and 100% respectively for IgG and 100% in both groups for IgA. In the SL cohorts, the 5 μg and 25 μg dmLT groups had response rates of 42% and 80% respectively for IgG and 17% and 60% respectively for IgA. Both IgG and IgA had 100% response rates in the 0.3 μg ID dmLT group (Supplementary Table 3).

#### 3.3.5 Linear mixed model analysis

There was a relationship between serum LT neutralizing antibody and both serum IgG and IgA ELISA responses. However, since there were few ASC and ALS results above the limits of detection (LOD), the linear mixed models demonstrated poor model fit for these data (Supplementary Tables 4–9).

There was a statistically significant relationship between serum LT neutralizing antibody and dmLT-specific serum IgA in the oral and ID routes. For the oral route, there is also a statistically significant interaction between serum LT neutralization and dose level with participants receiving 25 μg dmLT showing a larger increase in serum IgA for each 2-fold increase in serum LT neutralization titer than participants receiving 5 μg dmLT.

There was a statistically significant relationship between serum LT neutralizing antibody and dmLT-specific serum IgG for all administration routes. For both the oral and SL routes, there is also a statistically significant interaction between serum LT neutralization and dose level with participants receiving 25 μg dmLT showing a larger increase in serum IgG for each 2-fold increase in serum LT neutralization titer than participants receiving 5 μg dmLT.

The fixed effects in the serum IgG model explained more variation than those of serum IgA models (R_m_^2^ = 0.68 vs. 0.57 for oral, R_m_^2^ = 0.42 vs 0.04 for sublingual, and R_m_^2^ = 0.73 vs 0.71 for intradermal), and the fixed effects alone explained a higher proportion of the total variance than the random effects alone. The total variation explained by both the fixed and random effects were also higher for serum IgG and serum IgA in the oral (R_c_^2^ = 0.84 vs 0.82) and SL routes (R_c_^2^ = 0.71 vs 0.59) but not in the ID route (R_c_^2^ = 0.87 vs 0.88).

## 4 Discussion

This study was designed to measure immune responses to dmLT when administered by 2 mucosal routes, i.e., oral and SL, and by a systemic route, i.e., ID, in healthy adults who have presumably been previously immunologically primed through natural infections with ETEC due to their long-term residence in an ETEC endemic location. We demonstrated that dmLT when delivered through various routes of administration is safe, generally well-tolerated, and highly immunogenic by all three routes studied. No participants developed fever and fatigue in any of the three administration routes. Participants who developed systemic solicited AE (n=8) were mostly mild headache (6/8) and were observed after oral and sublingual administration routes. 3 participants vomited after oral doses (25 μg) and 2 participants showed site erythema after intradermal doses (Table 1). These solicited AE were very mild and suggests dmLT as safe and tolerable by three different routes. In relation to immunogenicity, 25 μg oral and 0.3 μg intradermal doses had shown best responses (Table 2; Figures 2–4) for all parameters (serum, ALS and ASC responses) in compared to other doses and routes and suggests dmLT as immunogenic.

While ETEC is well recognized as a major cause of diarrhea among infants and young children in LMICs, causing an estimated 75 million episodes and 18,000 to 42,000 deaths among children under the age of 5 years, the burden of disease among adults is less well characterized. A meta-analysis estimated that ETEC may cause 89,000 annual deaths among persons older than 5 years in LMICs. A high burden of severe ETEC diarrhea requiring hospitalization has been identified among adults 20–60 years of age in rural Bangladesh suggesting that there might also be a need to target preventative measures such as vaccines for adults (Chakraborty et al., 2024). Considering these high burden of ETEC diarrhea, this study is of great importance.

Site-directed mutagenesis consisting of a glycine substitution at position 192 by arginine (R192G) was performed to disrupt the toxigenic activity of the A subunit (Lycke et al., 1992; Dickinson and Clements, 1995), resulting in a single-mutant LT (mLT or LT (R192G) protein). In initial trials, 25 μg of LT(R192G) was associated with cases of mild, self-limited diarrhea when co-administered with other antigens (Kotloff et al., 2001; Lapa et al., 2008). A second-generation derivative, double-mutant LT (dmLT or LT(R192G/L211A)), was created through the additional substitution of alanine for leucine at amino acid position 211 (L211A) (Norton et al., 2011). In an initial Phase 1 trial in U.S. adults, single oral doses up to 100 μg dmLT demonstrated no diarrheal reactogenicity and was found to be immunogenic (El-Kamary et al., 2013). In further Phase 1 studies in U.S. adults, up to 50 μg of SL was administered (Bernstein et al., 2019), and up to 2 μg of ID doses of dmLT were observed to be safe and have dmLT dose-dependent immune responses [NCT02531685, unpublished].

Anti-LT reactive antibodies have also been shown to modulate the severity of ETEC associated illness following experimental infection (McKenzie et al., 2007; Gutiérrez et al., 2024) and to protect against ETEC strains producing only LT in field studies (Clemens et al., 1988; Behrens et al., 2014). Additionally, in the field, anti-LT antibodies have also been shown to reduce the severity of ETEC-associated disease in general (Frech et al., 2008; Behrens et al., 2014). Recent observations that the intestinal cAMP increase caused by LT can drive enteropathic changes in the gut that could contribute to the longer-term negative health effects of ETEC infection among infants and young children in LMICs, as well as triggering receptor express that may make infants and young children more susceptible to other enteric pathogens, further strengthen the rationale for including dmLT in ETEC and combination enteric vaccines under development (Glenn et al., 2009; Sheikh et al., 2020; Sheikh et al., 2022).

Although the study did not directly compare the immune responses between non-endemic (or immunologically naïve) and endemic (presumed immunologically previously primed) persons, some points can be made considering the findings from this study and data from two published reports of oral and SL dmLT in healthy U.S. adult participants (El-Kamary et al., 2013; Bernstein et al., 2019). U.S. adults demonstrated a plateau response to 50 μg of dmLT when administered orally and 25 μg of dmLT sublingually, as measured by serum ELISA seroconversion rates. Only 1 of 6 U.S. adults receiving a single oral dose of 25 μg dmLT (El-Kamary et al., 2013) and only 5 of 15 U.S. adults receiving three oral doses of 25 μg of dmLT (Bernstein et al., 2019) demonstrated serum anti-dmLT IgG seroconversions. In contrast, our study documented serum IgG seroconversions in 8 of 12 Bangladeshi adults after dose 1 and all 12 participants following three oral doses of 25 μg dmLT. Similar trends for seroconversion rates are observed for the elicitation of serum anti-dmLT IgA and toxin neutralizing antibody responses. Our linear mixed model analysis demonstrated statistically significant relationships between the elicitation of serum LT neutralizing (functional antibody) and serum IgG and IgA antibody responses.

The excellent serum and toxin neutralizing antibody response induced by the 25 μg oral dose and 0.3 μg ID doses also further highlight the potential value of dmLT as a safe antigen for inclusion in ETEC vaccines candidates and/or for inclusion in combination enteric vaccines that are being considered for development. The ALS responses demonstrated similar trends for the highest responses among the ID 0.3 μg and oral 25 μg groups with even higher responses after the second ID dose of dmLT. In both the ASC and ALS assays, the IgG responses were more prominent than the IgA responses. The trend was for the plasmablast responses to be most prevalent after the first dose of dmLT, when administered orally or SL, however the ID 0.3 μg group had an even higher proportion of responders after the second dose of dmLT. For individual assays (i.e., ASC, ALS, or ELISA assay), baseline responses were compared among different cohorts and different routes but no significant difference was observed in any of the cohorts or routes. This suggests that baseline responses were similar in participants before vaccination. We were unable to measure the immune response of Bangladeshi adults receiving higher oral or sublingual doses of dmLT (i.e., 50 μg). Overall, a dose of intradermal 0.3 μg and also a dose of oral 25 μg induced optimum mucosal and systemic dmLT-specific immune responses in most immunized individuals which are the highest dose tested. Based on these comparisons, these data appear to indicate that previously primed individuals can successfully respond to lower oral doses of dmLT and more vigorous responses were seen in an endemic population that are likely to have been primed with ETEC antigens in the past. In another mice model study, higher antibody responses were observed when dmLT was administered intradermally as an adjuvant with low protection in comparison to oral and sublingual routes (Luo et al., 2016). However, no such studies have been carried out in human.

The encouraging safety and immunogenicity of dmLT in adults through different routes support its further assessment for protective efficacy in adults and children in ETEC endemic areas. These encouraging data also support the potential addition of dmLT as an adjuvant and/or antigen in candidate vaccines, including multi-pathogen combination vaccines that may be developed for delivery by different routes. Our data show dmLT can be used to induce systemic immune responses to the ETEC vaccine antigens and the use of dmLT might improve these responses in adults in LMICs. The information gained in our study not only markedly advances the further development of ETEC vaccines but could also have important implications for more successful use of other oral vaccines, such as cholera where these concepts can be used.

This study has several advantages. Firstly, this study was carried out in a ETEC endemic setting. Secondly, we evaluated immune responses in three different routes to help determine the optimum route. Thirdly, dose escalation of dmLT was carried out for oral and SL routes. Apart from the evaluation of systemic immune response, mucosal immune responses from saliva and fecal samples and cellular responses were also evaluated and will be reported later.

Unfortunately, this study was interrupted by the COVID-19 pandemic which is the major limitation of the study. The timing of local shutdowns, due to the pandemic, prevented the evaluation of an originally planned three dose scheme of 0.3 μg ID dmLT, as well as, the original study plan to assess oral and SL 50 μg dmLT doses and ID 1.0 μg and 2.0 μg dmLT doses. The conclusion is limited by the fact that all the planned dose regimens could not be completed; it might be possible that additional sequential doses may elicit a higher response than the observed responses. A logical target group for this vaccine would be younger children and groups who have been difficult to effectively immunize with other oral vaccines. Successful completion of the planned doses could have created the opportunity to plan for future assessments of immune responses in children.

Collectively, this study demonstrated that all the doses of dmLT given by oral, sublingual, and intradermal routes, are safe and well tolerated. Immune responses are largely dependent on dmLT dose and route of administration. Based on our findings, we may summarize that systemic vaccination of dmLT may elicit better immune responses in comparison to mucosal vaccination in Bangladeshi adults, though, we cannot infer this hypothesis completely as the study was interrupted due to the COVID-19 pandemic. Nevertheless, further such dose escalating studies are required to determine the optimal route and dose of dmLT. However, these encouraging safety and immunogenicity results may serve to highlight the idea of inclusion of dmLT in new ETEC vaccines or with other enteric vaccine candidates.

## Supplementary Material

Supplemental Data

The Supplementary Material for this article can be found online at: https://www.frontiersin.org/articles/10.3389/fbrio.2025.1567791/full#supplementary-material

## Figures and Tables

**Table 1:** Occurrence of any reactogenicity during the 7 days post-vaccination, after any dose of vaccine. Number of participants with any respective symptom for each group is shown by the total number of participants in that group.

	Oral 5 μg dmLT (n=12)	Oral 25 μg dmLT (n=12)	All Oral Cohorts: Placebo (n=6)	Sublingual 5 μg dmLT (n=12)	Sublingual 25 μg dmLT (n=12)	All Sublingual Cohorts: Placebo (n=6)	Intradermal 0.3 μg dmLT (n=12)	Intradermal Placebo (n=3)
	
Systemic								
Fever	0	0	0	0	0	0	0	0
Fatigue	0	0	0	0	0	0	0	0
Malaise	1	0	0	0	0	0	0	0
Myalgia	2	0	0	1	0	0	0	0
Headache	2	1	0	2	0	1	0	0
								
Local								
Diarrhea	0	0	0	0	0	0	0	0
Nausea	0	0	0	0	0	0	0	0
Vomiting	0	3	0	0	0	0	0	0
Abdominal discomfort	0	0	0	0	0	0	0	0
Oral irritation^1^	-	-	-	0	0	0	-	-
Facial nerve^1^	-	-	-	0	0	0	-	-
Site pain^2^	-	-	-	-	-	-	0	0
Site erythema^2^	-	-	-	-	-	-	2	0
Site induration^2^	-	-	-	-	-	-	1	0
Site ecchymoses, pruritus, pigmentation, vesicles^2^	-	-	-	-	-	-	0	0

1 – irritation of the oral cavity or tongue and facial nerve disturbance were assessed for SL route only

2 – injection site pain, erythema, induration, ecchymoses, pruritus, pigmentation (hyper or hypopigmentation) or vesicles were assessed for the ID route only

**Table 2:** Serum LT Neutralizing Responses GMT (95% CI), by Study Group

	Oral 5 μg dmLT (n=12)	Oral 25 μg dmLT (n=12)	All Oral Cohorts: Placebo (n=6)	Sublingual 5 μg dmLT (n=12)	Sublingual 25 μg dmLT (n=10)	All Sublingual Cohorts: Placebo (n=6)	Intradermal 0.3 μg dmLT (n=12)	Intradermal Placebo (n=3)
	
Day 1 (baseline)	16.0 (7.7, 33.1)	24.0 (13.5, 42.7)	25.4 (7.1, 90.8)	13.5 (9.7, 18.8)	13.0 (7.3, 23.1)	18.0 (10.4, 31.1)	24.0 (14.4, 40.0)	20.2 (1.5, 279.7)
Day 8	40.3 (17.3, 94.0)	90.5 (39.5, 207.4)	18.0 (7.7, 42.0)	12.0 (8.1, 17.8)	10.6 (5.6, 19.8)	18.0 (10.4, 31.1)	30.2 (19.5, 46.8)	20.2 (1.5, 279.7)
Day 15/22	53.8 (19.9, 145.7)	256.0 (108.3, 605.3)	22.6 (7.5, 68.2)	14.3 (9.9, 20.6)	16.0 (8.3, 31.0)	18.0 (10.4, 31.1)	114.0 (52.8, 246.4)	25.4 (1.8, 352.4)
Day 22/29	76.1 (28.1, 206.0)	322.5 (135.5, 767.8)	20.2 (7.5, 54.5)	13.5 (9.2, 19.7)	17.1 (9.9, 29.6)	18.0 (10.4, 31.1)	143.7 (68.1, 303.3)	16.0 (0.8, 315.8)
Day 29/43	76.1 (21.6, 268.7)	362.0 (165.0, 794.2)	18.0 (6.2, 52.4)	15.1 (9.4, 24.3)	18.4 (7.7, 43.8)	16.0 (8.3, 30.7)	N/A	N/A
Day 36/50	85.4 (37.3, 195.6)	383.6 (179.1, 821.7)	20.2 (7.5, 54.5)	14.3 (9.1, 22.4)	26.0 (12.0, 56.5)	18.0 (7.7, 42.0)	N/A	N/A
Day 57/71	85.4 (37.3, 195.6)	287.4 (139.4, 592.3)	20.2 (7.5, 54.5)	11.7 (8.0, 17.1)	17.1 (9.5, 31.0)	20.2 (11.1, 36.5)	N/A	N/A
Day 114/128	53.8 (21.4, 135.4)	191.8 (114.8, 320.3)	20.2 (7.5, 54.5)	14.1 (8.5, 23.3)	N/A	20.2 (7.5, 54.5)	N/A	N/A
	
Total No (%) Responders*	11 (92)	12 (100)	0	0	2 (20)	0	10 (83)	0

* Responders are defined as achieving a 4-fold increase in neutralizing antibody over baseline, at any time post-vaccination.

N/A = Not Applicable. Day 15, 22, 29, 36, 57 and 114 are for oral and SL cohorts, and Day 22, 29, 43, 50, 71 and 128 are for ID cohort.

## Data Availability

The raw data supporting the conclusions of this article will be made available by the authors, without undue reservation.

## Abbreviations:

dmLT: Double-mutant heat-labile enterotoxin
ETEC: Enterotoxigenic *Escherichia coli*
ASC: Antibody secreting cell
ALS: Antibodies in lymphocyte supernatant
ST: Heat-stable toxin
LT: Heat-labile toxin
cAMP: Cyclic adenosine monophosphate
LMIC: Low- and middle-income countries
ID: Intradermal
IM: Intramuscular
SL: Sublingual
cGMP: current Good Manufacturing Practices
ELISA: Enzyme-linked immunosorbent assay
ELISpot: Enzyme-linked immunosorbent spot
AE: Adverse events
GMT: Geometric mean titer
CHIM: Controlled human infection model
PBMC: Peripheral Blood Mononuclear Cell
OD: Optical Density
SFC: Spot Forming Cells
mITT: modified Intention to Treat
