# Supplementary material for: Safety and immunogenicity of a recombinant double-mutant heat-labile toxin derived from enterotoxigenic Escherichia coli in healthy Bangladeshi adults delivered by three different routes
Source: Front Bacteriol. Author manuscript; Available in PMC 2025 Nov 26. (PMC12646041; doi:10.3389/fbrio.2025.1567791)
Supplement: Supplemental Data [file NIHMS2123077-supplement-Supplemental_Data.docx]

Supplementary Material

Safety and Immunogenicity of a Recombinant Double-Mutant Heat-Labile Toxin (dmLT) Derived from Enterotoxigenic *Escherichia coli* (ETEC) in Healthy Bangladeshi Adults delivered by three different routes

**Taufiqur Rahman Bhuiyan^1^, Farhana Khanam^1^, Salima Raiyan Basher^1^, Pinki Dash^1^, Mohiul Islam Chowdhury^1^, Shahinur Haque^1^, Nabila Binte Harun^1^, Aklima Akter^1^, Polash Chandra Karmakar^1^, Al Hakim^1^, Shaheena Amin^1^, Mohammad Kamruzzaman^1^, Nasrin Parvin^1^, Tasnuva Ahmed^1^, Jessica Butts^2^, Marcela F. Pasetti^3^, Rezwanul Wahid^3^, Marcelo B. Sztein^3^, Nicole Maier^4^, Jessica A. White^4^, Kay Tomashek^5^, A. Louis Bourgeois^4^, Shahida Baqar^5^, Karen L. Kotloff^3^**^†^**, Firdausi Qadri^1^**^†^**, Wilbur H. Chen^3^**^†^**^*^**

*** Correspondence:**

Wilbur H. Chen, M.D., M.S. ([Wilbur.Chen@som.umaryland.edu](mailto:Wilbur.Chen@som.umaryland.edu))

**Supplemental Table 1**: Serum IgA and IgG Responses (GMT and 95% CI), by Study Group

|  | | **Oral 5 µg dmLT (n=12)** | **Oral 25 µg dmLT (n=12)** | **All Oral Cohorts: Placebo (n=6)** | **Sublingual 5 µg dmLT (n=12)** | | **Sublingual 25 µg dmLT (n=10)** | **All Sublingual Cohorts: Placebo (n=6)** | **Intradermal 0.3 µg dmLT (n=12)** | **Intradermal Placebo (n=3)** |
| --- | --- | --- | --- | --- | --- | --- | --- | --- | --- | --- |
| Anti-dmLT Serum IgA (EU/mL) | | | | | | | | | | |
| Day 1 | | 311 (247, 393) | 287 (219, 377) | 292 (200, 427) | 313 (215, 455) | | 249 (150, 414) | 222 (67.2, 733) | 455 (333, 622) | 253 (25.4, 2521) |
| Day 8 | | 337 (258, 441) | 852 (496, 1463) | 252 (179, 357) | 302 (198, 461) | | 260 (164, 411) | 210 (99, 445) | 484 (346, 676) | 218 (33.6, 1413) |
| Day 15/22 | | 386 (249, 598) | 1416 (820, 2446) | 265 (176, 398) | 261 (165, 411) | | 230 (142, 375) | 189 (63.6, 562) | 3160 (1673, 5967) | 274 (40.6, 1845) |
| Day 22/29 | | 423 (288, 622) | 1509 (1002, 2271) | 265 (175, 404) | 268 (184, 391) | | 309 (176, 543) | 182 (62.9, 526) | 3344 (1838, 6084) | 241 (39.7, 1465) |
| Day 29/43 | | 424 (268, 671) | 1385 (848, 2262) | 300 (158, 568) | 367 (250, 540) | | 343 (170, 694) | 183 (63.1, 532) | N/A | N/A |
| Day 36/50 | | 371 (249, 553) | 1644 (1010, 2677) | 278 (171, 453) | 301 (202, 449) | | 347 (195, 618) | 171 (65.3, 447) | N/A | N/A |
| Day 57/71 | | 338 (247, 462) | 1004 (617, 1634) | 331 (198, 555) | 304 (194, 477) | | 307 (159, 592) | 175 (70.2, 438) | N/A | N/A |
| Day 114/128 | | 287 (218, 377) | 502 (318, 791) | 295 (200, 437) | 324 (210, 498) | | N/A | 141 (8.4, 2374) | N/A | N/A |
| Total No. (%) Responders* | 2 (17) | 11 (92) | 0 | 0 | | 2 (20) | 0 | 10 (83) | 0 |  |
|  |  |  | Anti-dmLT Serum IgG (EU/mL) | |  |  |  |  |  |  |
| Day 1 | | 3805  (2391, 6056) | 3260  (1817, 5850) | 3341  (1969, 5671) | 2242  (1295, 3880) | | 2902  (1594, 5282) | 3442  (1826, 6487) | 3450  (2364, 5037) | 4492  (513, 39353) |
| Day 8 | | 4995  (2900, 8605) | 7021  (3545, 13905) | 3105  (1704, 5658) | 2065  (1213, 3514) | | 2928  (1534, 5587) | 2961  (1648, 5322) | 3741  (2679, 5225) | 4313  (486, 38302) |
| Day 15/22 | | 6860  (3157, 14910) | 19558  (9155, 41781) | 2916  (1617, 5258) | 1962  (1162, 3311) | | 3088  (1687, 5654) | 2861  (1509, 5425) | 23693  (13276, 42283) | 4268  (418, 43568) |
| Day 22/29 | | 8620  (3842, 19340) | 23586  (11343, 49041) | 2933  (1606, 5357) | 1937  (1110, 3381) | | 4036  (2181, 7466) | 3050  (1796, 5179) | 27172  (15821, 46669) | 4692  (471, 46715) |
| Day 29/43 | | 10007  (4668, 21454) | 27326  (13837, 53965) | 2477  (1148, 5345) | 2193  (1302, 3696) | | 5674  (2773, 11610) | 3223  (1832, 5671) | N/A | N/A |
| Day 36/50 | | 9633  (5138, 1806) | 27132  (13900, 52963) | 2149  (1070, 4316) | 2036  (1146, 3618) | | 5019  (2587, 9737) | 3021  (1756, 5198) | N/A | N/A |
| Day 57/71 | | 11457  (6801, 19303) | 25301  (14064, 45517) | 2273  (1020, 5069) | 1695  (1004, 2859) | | 5169  (2706, 9873) | 2962  (1762, 4982) | N/A | N/A |
| Day 114/128 | | 9049  (6055, 13524) | 13183  (6993, 24854) | 2275  (1029, 5030) | 1739  (1015, 2981) | | N/A | 4489  (1878, 10731) | N/A | N/A |
| Total No. (%) Responders* | | 6 (50) | 12 (100) | 0 | 0 | | 2 (20) | 0 | 9 (75) | 0 |
| *Responders are defined as achieving a 4-fold increase in antibody over baseline, at any time post-vaccination; N/A = Not Applicable. Day 15, 22, 29, 36, 57 and 114 are for oral and SL cohorts, and Day 22, 29, 43, 50, 71 and 128 are for ID cohort. | | | | | | | | | | |

**Supplemental Table 2**: Antibody secreting cell (ASC) responses, by Study Group

|  | **Oral 5 µg dmLT (n=12)** | **Oral 25 µg dmLT (n=12)** | **All Oral Cohorts: Placebo (n=6)** | **Sublingual**  **5 µg dmLT (n=12)** | **Sublingual 25 µg dmLT (n=10)** | **All Sublingual Cohorts: Placebo (n=6)** | **Intradermal 0.3 µg dmLT (n=12)** | **Intradermal**  **Placebo (n=3)** |  |  |
| --- | --- | --- | --- | --- | --- | --- | --- | --- | --- | --- |
| Number (%) of ASC IgG responders | | | | | | | | | | |
| Pre-dose 1 | | | 0 | 0 | 0 | 0 | 1 (10) | 0 | 0 | 0 |
| 7-days post-dose 1 | | | 3 (25) | 7 (58) | 0 | 0 | 2 (20) | 1 (17) | 7 (58) | 0 |
| Pre-dose 2 | | | 0 | 0 | 0 | 0 | 1 (10) | 0 | 2 (17) | 0 |
| 7-days post-dose 2 | | | 2 (17) | 2 (17) | 0 | 1 (8) | 2 (20) | 0 | 10 (83) | 0 |
| Pre-dose 3 | | | 0 | 0 | 0 | 1 (8) | 1 (10) | 0 | N/A | N/A |
| 7-days post-dose 3 | | | 1 (8) | 2 (17) | 0 | 0 | 1 (10) | 0 | N/A | N/A |
| Total No. (%)  Responders* | | | 5 (50) | 8 (67) | 0 | 2 (17) | 3 (30) | 1 (17) | 10 (83) | 0 |
| Number (%) of ASC IgA responders | | | | | | | | | | |
| Pre-dose 1 | | 0 | 1 (8) | 0 | 0 | 0 | 0 | 0 | 0 |  |
| 7-days post-dose 1 | | 0 | 3 (25) | 0 | 1 (8) | 0 | 0 | 2 (17) | 0 |  |
| Pre-dose 2 | | 0 | 0 | 0 | 0 | 0 | 0 | 0 | 0 |  |
| 7-days post-dose2 | | 0 | 1 (8) | 0 | 0 | 0 | 0 | 5 (42) | 0 |  |
| Pre-dose 3 | | 0 | 0 | 0 | 0 | 0 | 0 | N/A | N/A |  |
| 7-days post-dose 3 | | 0 | 0 | 0 | 0 | 0 | 0 | N/A | N/A |  |
| Total No. (%)  Responders* | | 0 | 3 (25) | 0 | 1 (8) | 0 | 0 | 5 (42) | 0 |  |
| *Responders are defined as ≥8 spot forming cells/10^6^ PBMC at any time post-dose 1; N/A = Not Applicable. | | | | | | | | | |  |

**Supplemental Table 3**: Antibodies in lymphocyte supernatants (ALS) responses, by Study Group

| **7 days post-dose** | **Oral 5 µg**  **dmLT (n=12)** | **Oral 25 µg dmLT (n=12)** | **All Oral Placebo (n=6)** | **Sublingual**  **5 µg dmLT (n=12)** | **Sublingual**  **25 µg dmLT (n=10)** | **All Sublingual Placebo (n=6)** | **Intradermal**  **0.3 µg dmLT (N=12)** | **Intradermal Placebo (n=3)** |
| --- | --- | --- | --- | --- | --- | --- | --- | --- |
| ALS IgG: GMFR (95% CI) % responders | | | | | | | | |
| 1 | 13.5 (3.9, 47.5) 67 | 73.2 (30.0, 178.3)  100 | 1.2 (0.7, 2.1)  17 | - 1. (0.9, 1.5)   8 | 1.4 (0.5, 4.0)  40 | 0.8 (0.5, 1.2)  0 | 34.4 (13.4, 88.5)  100 | 0.6 (0.1, 4.2)  0 |
| 2 | 4.4 (1.5, 12.6)  50 | 6.8 (3.2, 14.3)  83 | - 1. (0.8, 1.5)   0 | 1.7 (0.9, 3.4)  25 | 4.8 (1.6, 14.1)  60 | 0.8 (0.5, 1.2)  0 | 127.2 (53.5, 302.4)  100 | 0.6 (0.1, 4.2)  0 |
| 3 | 2.0 (0.7, 5.7)  42 | 5.5 (1.9, 16.5)  58 | 1. (NC)   0 | 1.4 (0.8, 2.5)  17 | 1.4 (0.5, 3.9)  30 | 1.3 (0.4, 4.1)  17 | N/A | N/A |
| *Responder  n (%) | 11 (92) | 12 (100) | 2 (33) | 5 (42) | 8 (80) | 1 (17) | 12 (100) | 1(33) |
| ALS IgA: GMFR (95% CI) % responders | | | | | | | | |
| 1 | 5.1 (2.4, 10.9)  75 | 36.2 (16.3, 80.4)  100 | 1. (NC)   0 | 1. (1.0, 1.1)   0 | 0.9 (0.4, 1.9)  10 | 1. (NC)   0 | 8.3 (3.3, 21.2)  83 | 0.8 (0.2, 2.5)  0 |
| 2 | 2.0 (0.9, 4.4)  42 | 3.4 (1.6, 7.2)  42 | 1. (NC)   0 | 1.1 (0.9, 1.4)  8 | 2.3 (0.7, 7.2)  40 | 1.4 (0.6, 3.5)  17 | 31.9 (11.0, 92.7)  92 | 0.8 (0.2, 2.5)  0 |
| 3 | - 1. (0.9, 2.4)   25 | 3.9 (1.5, 10.2)  42 | 1. (1.0, 1.0)   0 | 1.1 (0.8, 1.7)  8 | 1.5 (0.6, 3.7)  30 | - 1. (0.7, 2.0)   17 | N/A | N/A |
| *Responder n (%) | 12 (100) | 12 (100) | 1 (17) | 2 (17) | 6 (60) | 2 (33) | 12 (100) | 0 |
| GMFR, geometric mean fold rise above baseline  NC = not calculable; N/A = Not Applicable.  *Responders are defined as ≥2-fold increase in titer over baseline, at any time post-dose 1 | | | | | | | | |

**Supplemental Table 4:** Linear Mixed Model Results for Serum IgA in Participants Receiving dmLT by Administration Route, mITT Population

|  | Oral Administration (N=24, Nobs=192) | | Sublingual Administration (N=22, Nobs=164) | | | | Intradermal Administration (N=12, Nobs=48) | |
| --- | --- | --- | --- | --- | --- | --- | --- | --- |
|  | Interaction Model R_m_^2^ = 0.57  R_c_^2^ = 0.82 | | Interaction Model R_m_^2^ = 0.05  R_c_^2^ = 0.60 | | Main Effects Model R_m_^2^ = 0.04  R_c_^2^ = 0.59 | | Main Effect Model R_m_^2^ = 0.71  R_c_^2^ = 0.88 | |
| Fixed Effect | Est (SE) | P-value^a^ | Est (SE) | P-value^a^ | Est (SE) | P-value^a^ | Est (SE) | P-value^a^ |
| Intercept | 7.62 (0.32) | N/A | 7.98 (0.43) | N/A | 7.57 (0.53) | N/A | 5.53 (0.44) | N/A |
| Log_2_ Serum LT Neut | 0.15 (0.05) | <0.001 | 0.06 (0.08) | 0.134 | 0.17 (0.12) | 0.151 | 0.81 (0.07) | <0.001 |
| Dose = 25 µg dmLT | -1.60 (0.45) | <0.001 | -0.70 (0.74) | 0.346 | -0.09 (0.41) | 0.823 | N/A | N/A |
| Log_2_ Serum LT Neut * Dose = 25 µg dmLT | 0.36 (0.07) | <0.001 | 0.16 (0.19) | 0.402 | N/A | N/A | N/A | N/A |
| Notes: N = Number of participants included in the model; Nobs = Number of participant-visits included in the model; Est = Estimate; SE = Standard Error; N/A = Not Applicable. Results are from a linear mixed model with the fixed effects listed in the table and a random intercept for participant; all models are estimated using restricted maximum likelihood. R_m_^2^ and R_c_^2^ are the marginal and conditional R^2^ values described by Nakagawa and Schielzeth (2013) and represent the proportion of variance explained by the fixed effects and by both the fixed and random effects respectively. Serum IgA responses are log_2_-transformed. Models include participants in the mITT Population who received dmLT; placebo participants are excluded. ^a^ P-values are from Type 3 Tests of Fixed Effects based on an F statistic. | | | | | | | | |

**Supplemental Table 5:** Linear Mixed Model Results for Serum IgG in Participants Receiving dmLT by Administration Route, mITT Population

|  | Oral Administration (N=24, Nobs=192) | | Sublingual Administration (N=22, Nobs=164) | | Intradermal Administration (N=12, Nobs=48) | |
| --- | --- | --- | --- | --- | --- | --- |
|  | Interaction Model R_m_^2^ = 0.68  R_c_^2^ = 0.84 | | Interaction Model R_m_^2^ = 0.42  R_c_^2^ = 0.71 | | Main Effect Model R_m_^2^ = 0.73  R_c_^2^ = 0.87 | |
| Fixed Effect | Est (SE) | P-value^a^ | Est (SE) | P-value^a^ | Est (SE) | P-value^a^ |
| Intercept | 10.43 (0.70) | N/A | 10.14 (0.46) | N/A | 8.23 (0.35) | N/A |
| Log_2_ Serum LT Neut | 0.43 (0.10) | <0.001 | 0.23 (0.09) | <0.001 | 0.85 (0.07) | <0.001 |
| Dose = 25 µg dmLT | -2.05 (0.93) | 0.029 | -0.94 (1.00) | 0.346 | N/A | N/A |
| Log_2_ Serum LT Neut * Dose = 25 µg dmLT | 0.30 (0.12) | 0.016 | 0.46 (0.23) | 0.048 | N/A | N/A |
| Notes: N = Number of participants included in the model; Nobs = Number of participant-visits included in the model; Est = Estimate; SE = Standard Error; N/A = Not Applicable. Results are from a linear mixed model with the fixed effects listed in the table and a random intercept for participant; all models are estimated using restricted maximum likelihood. R_m_^2^ and R_c_^2^ are the marginal and conditional R^2^ values described by Nakagawa and Schielzeth (2013) and represent the proportion of variance explained by the fixed effects and by both the fixed and random effects respectively. Serum IgG responses are log_2_-transformed. Models include participants in the mITT Population who received dmLT; placebo participants are excluded. ^a^ P-values are from Type 3 Tests of Fixed Effects based on an F statistic. | | | | | | |

**Supplemental Table 6:** Linear Mixed Model Results for ALS IgA in Participants Receiving dmLT by Administration Route, mITT Population

|  | Oral Administration (N=24, Nobs=144) | | | | Sublingual Administration (N=22, Nobs=132) | | | | Intradermal Administration (N=12, Nobs=48) | |
| --- | --- | --- | --- | --- | --- | --- | --- | --- | --- | --- |
|  | Interaction Model R_m_^2^ = 0.05  R_c_^2^ = 0.05 | | Main Effects Model R_m_^2^ = 0.05  R_c_^2^ = 0.05 | | Interaction Model R_m_^2^ = 0.04  R_c_^2^ = 0.04 | | Main Effects Model R_m_^2^ = 0.04  R_c_^2^ = 0.04 | | Main Effect Model R_m_^2^ = 0.09  R_c_^2^ = 0.09 | |
| Fixed Effect | Est (SE) | P-value^a^ | Est (SE) | P-value^a^ | Est (SE) | P-value^a^ | Est (SE) | P-value^a^ | Est (SE) | P-value^a^ |
| Intercept | 0.69 (0.17) | N/A | 0.70 (0.23) | N/A | 0.36 (0.43) | N/A | -0.14 (0.60) | N/A | 1.29 (0.83) | N/A |
| Log_2_ Serum LT Neut | 0.07 (0.03) | 0.063 | 0.07 (0.04) | 0.063 | 0.05 (0.11) | 0.247 | 0.18 (0.16) | 0.242 | 0.34 (0.12) | 0.007 |
| Dose = 25 µg dmLT | 0.72 (0.51) | 0.161 | 0.70 (0.16) | <0.001 | -0.17 (0.79) | 0.833 | 0.56 (0.35) | 0.111 | N/A | N/A |
| Log_2_ Serum LT Neut * Dose = 25 µg dmLT | -0.00 (0.08) | 0.964 | N/A | N/A | 0.19 (0.25) | 0.445 | N/A | N/A | N/A | N/A |
| Notes: N = Number of participants included in the model; Nobs = Number of participant-visits included in the model; Est = Estimate; SE = Standard Error; N/A = Not Applicable. Results are from a linear mixed model with the fixed effects listed in the table and a random intercept for participant; all models are estimated using restricted maximum likelihood. R_m_^2^ and R_c_^2^ are the marginal and conditional R^2^ values described by Nakagawa and Schielzeth (2013) and represent the proportion of variance explained by the fixed effects and by both the fixed and random effects respectively. ALS IgA responses are log_2_-transformed. Models include participants in the mITT Population who received dmLT; placebo participants are excluded. ^a^ P-values are from Type 3 Tests of Fixed Effects based on an F statistic. | | | | | | | | | | |

**Supplemental Table 7:** Linear Mixed Model Results for ALS IgG in Participants Receiving dmLT by Administration Route, mITT Population

|  | Oral Administration (N=24, Nobs=144) | | Sublingual Administration (N=22, Nobs=132) | | | | Intradermal Administration (N=12, Nobs=48) | |
| --- | --- | --- | --- | --- | --- | --- | --- | --- |
|  | Interaction Model R_m_^2^ = 0.14  R_c_^2^ = 0.14 | | Interaction Model R_m_^2^ = 0.04  R_c_^2^ = 0.04 | | Main Effects Model R_m_^2^ = 0.04  R_c_^2^ = 0.04 | | Main Effect Model R_m_^2^ = 0.24  R_c_^2^ = 0.24 | |
| Fixed Effect | Est (SE) | P-value^a^ | Est (SE) | P-value^a^ | Est (SE) | P-value^a^ | Est (SE) | P-value^a^ |
| Intercept | 0.85 (0.44) | N/A | -0.06 (0.54) | N/A | 0.73 (0.90) | N/A | 0.28 (0.74) | N/A |
| Log_2_ Serum LT Neut | 0.18 (0.07) | <0.001 | 0.21 (0.16) | 0.739 | -0.00 (0.24) | 0.993 | 0.73 (0.13) | <0.001 |
| Dose = 25 µg dmLT | -1.37 (0.67) | 0.044 | 2.08 (1.43) | 0.150 | 0.94 (0.42) | 0.027 | N/A | N/A |
| Log_2_ Serum LT Neut * Dose = 25 µg dmLT | 0.28 (0.10) | 0.006 | -0.30 (0.35) | 0.399 | N/A | N/A | N/A | N/A |
| Notes: N = Number of participants included in the model; Nobs = Number of participant-visits included in the model; Est = Estimate; SE = Standard Error; N/A = Not Applicable. Results are from a linear mixed model with the fixed effects listed in the table and a random intercept for participant; all models are estimated using restricted maximum likelihood. R_m_^2^ and R_c_^2^ are the marginal and conditional R^2^ values described by Nakagawa and Schielzeth (2013) and represent the proportion of variance explained by the fixed effects and by both the fixed and random effects respectively. ALS IgG responses are log_2_-transformed. Models include participants in the mITT Population who received dmLT; placebo participants are excluded. ^a^ P-values are from Type 3 Tests of Fixed Effects based on an F statistic. | | | | | | | | |

**Supplemental Table 8:** Linear Mixed Model Results for ASC IgA in Participants Receiving dmLT by Administration Route, mITT Population

|  | Oral Administration (N=24, Nobs=144) | | | | Sublingual Administration (N=22, Nobs=132) | | | | Intradermal Administration (N=12, Nobs=48) | |
| --- | --- | --- | --- | --- | --- | --- | --- | --- | --- | --- |
|  | Interaction Model R_m_^2^ = 0.05  R_c_^2^ = 0.05 | | Main Effects Model R_m_^2^ = 0.05  R_c_^2^ = 0.05 | | Interaction Model R_m_^2^ = 0.00  R_c_^2^ = 0.00 | | Main Effects Model R_m_^2^ = 0.00  R_c_^2^ = 0.00 | | Main Effect Model R_m_^2^ = 0.22  R_c_^2^ = 0.22 | |
| Fixed Effect | Est (SE) | P-value^a^ | Est (SE) | P-value^a^ | Est (SE) | P-value^a^ | Est (SE) | P-value^a^ | Est (SE) | P-value^a^ |
| Intercept | 0.06 (0.08) | N/A | 0.10 (0.16) | N/A | 0.07 (0.16) | N/A | 0.16 (0.15) | N/A | -0.64 (0.42) | N/A |
| Log_2_ Serum LT Neut | 0.01 (0.01) | 0.943 | 0.00 (0.03) | 0.943 | 0.04 (0.04) | 0.568 | 0.01 (0.04) | 0.737 | 0.25 (0.06) | <0.001 |
| Dose = 25 µg dmLT | 0.48 (0.42) | 0.252 | 0.40 (0.13) | 0.002 | 0.15 (0.24) | 0.542 | 0.00 (0.10) | 0.970 | N/A | N/A |
| Log_2_ Serum LT Neut * Dose = 25 µg dmLT | -0.01 (0.06) | 0.824 | N/A | N/A | -0.04 (0.07) | 0.570 | N/A | N/A | N/A | N/A |
| Notes: N = Number of participants included in the model; Nobs = Number of participant-visits included in the model; Est = Estimate; SE = Standard Error; N/A = Not Applicable. Results are from a linear mixed model with the fixed effects listed in the table and a random intercept for participant; all models are estimated using restricted maximum likelihood. R_m_^2^ and R_c_^2^ are the marginal and conditional R^2^ values described by Nakagawa and Schielzeth (2013) and represent the proportion of variance explained by the fixed effects and by both the fixed and random effects respectively. ASC IgA responses are log_2_-transformed. Models include participants in the mITT Population who received dmLT; placebo participants are excluded. ^a^ P-values are from Type 3 Tests of Fixed Effects based on an F statistic. | | | | | | | | | | |

**Supplemental Table 9:** Linear Mixed Model Results for ASC IgG in Participants Receiving dmLT by Administration Route, mITT Population

|  | Oral Administration (N=24, Nobs=144) | | | | Sublingual Administration (N=22, Nobs=132) | | | | Intradermal Administration (N=12, Nobs=48) | |
| --- | --- | --- | --- | --- | --- | --- | --- | --- | --- | --- |
|  | Interaction Model R_m_^2^ = 0.06  R_c_^2^ = 0.06 | | Main Effects Model R_m_^2^ = 0.06  R_c_^2^ = 0.06 | | Interaction Model R_m_^2^ = 0.03  R_c_^2^ = 0.03 | | Main Effects Model R_m_^2^ = 0.03  R_c_^2^ = 0.03 | | Main Effect Model R_m_^2^ = 0.25  R_c_^2^ = 0.25 | |
| Fixed Effect | Est (SE) | P-value^a^ | Est (SE) | P-value^a^ | Est (SE) | P-value^a^ | Est (SE) | P-value^a^ | Est (SE) | P-value^a^ |
| Intercept | -0.01 (0.15) | N/A | -0.19 (0.21) | N/A | -0.30 (0.29) | N/A | 0.40 (0.40) | N/A | -0.63 (0.80) | N/A |
| Log_2_ Serum LT Neut | 0.10 (0.02) | <0.001 | 0.13 (0.03) | <0.001 | 0.16 (0.10) | 0.706 | -0.03 (0.11) | 0.801 | 0.48 (0.14) | 0.001 |
| Dose = 25 µg dmLT | -0.17 (0.42) | 0.682 | 0.25 (0.17) | 0.157 | 1.49 (0.66) | 0.026 | 0.48 (0.32) | 0.131 | N/A | N/A |
| Log_2_ Serum LT Neut * Dose = 25 µg dmLT | 0.06 (0.06) | 0.281 | N/A | N/A | -0.26 (0.16) | 0.096 | N/A | N/A | N/A | N/A |
| Notes: N = Number of participants included in the model; Nobs = Number of participant-visits included in the model; Est = Estimate; SE = Standard Error; N/A = Not Applicable. Results are from a linear mixed model with the fixed effects listed in the table and a random intercept for participant; all models are estimated using restricted maximum likelihood. R_m_^2^ and R_c_^2^ are the marginal and conditional R^2^ values described by Nakagawa and Schielzeth (2013) and represent the proportion of variance explained by the fixed effects and by both the fixed and random effects respectively. ASC IgG responses are log_2_-transformed. Models include participants in the mITT Population who received dmLT; placebo participants are excluded. ^a^ P-values are from Type 3 Tests of Fixed Effects based on an F statistic. | | | | | | | | | | |
